# Internet of Medical Things and Healthcare 4.0: Trends, Requirements, Challenges, and Research Directions

**DOI:** 10.3390/s23177435

**Published:** 2023-08-25

**Authors:** Manar Osama, Abdelhamied A. Ateya, Mohammed S. Sayed, Mohamed Hammad, Paweł Pławiak, Ahmed A. Abd El-Latif, Rania A. Elsayed

**Affiliations:** 1 Department of Electronics and Communications Engineering, Zagazig University, Zagazig 44519, Egypt; m.osama21@eng.zu.edu.eg (M.O.); mohammed.sayed@ejust.edu.eg (M.S.S.); rania_ahmed@zu.edu.eg (R.A.E.); 2 EIAS Data Science Lab, College of Computer and Information Sciences, Prince Sultan University, Riyadh 11586, Saudi Arabia; mhammad@psu.edu.sa (M.H.); aabdellatif@psu.edu.sa (A.A.A.E.-L.); 3 Department of Electronics and Communication Engineering, Egypt-Japan University of Science and Technology, Alexandria 21934, Egypt; 4 Department of Information Technology, Faculty of Computers and Information, Menoufia University, Shibin El Kom 32511, Egypt; 5 Department of Computer Science, Faculty of Computer Science and Telecommunications, Cracow University of Technology, Warszawska 24, 31-155 Krakow, Poland; 6 Institute of Theoretical and Applied Informatics, Polish Academy of Sciences, Bałtycka 5, 44-100 Gliwice, Poland; 7 Department of Mathematics and Computer Science, Faculty of Science, Menoufia University, Shibin El Kom 32511, Egypt

**Keywords:** Internet of Medical Things, Healthcare 4.0, artificial intelligence, distributed edge computing, 5G, e-health

## Abstract

Healthcare 4.0 is a recent e-health paradigm associated with the concept of Industry 4.0. It provides approaches to achieving precision medicine that delivers healthcare services based on the patient’s characteristics. Moreover, Healthcare 4.0 enables telemedicine, including telesurgery, early predictions, and diagnosis of diseases. This represents an important paradigm for modern societies, especially with the current situation of pandemics. The release of the fifth-generation cellular system (5G), the current advances in wearable device manufacturing, and the recent technologies, e.g., artificial intelligence (AI), edge computing, and the Internet of Things (IoT), are the main drivers of evolutions of Healthcare 4.0 systems. To this end, this work considers introducing recent advances, trends, and requirements of the Internet of Medical Things (IoMT) and Healthcare 4.0 systems. The ultimate requirements of such networks in the era of 5G and next-generation networks are discussed. Moreover, the design challenges and current research directions of these networks. The key enabling technologies of such systems, including AI and distributed edge computing, are discussed.

## 1. Introduction

There is a great demand for digitalizing healthcare sectors, especially with the advancements in wireless technologies and electronic devices. Healthcare 4.0 is the latest paradigm in healthcare systems incorporating modern technologies, including the Internet of Things (IoT), artificial intelligence (AI), and cloud computing [1,2]. It refers to adapting health-management systems, which can be achieved via cloud computing and mobile communications. Clinical images can be continuously evaluated with greater precision and accuracy through digitalization [3].

Quality healthcare is a fundamental human right; it is not always provided adequately worldwide. Chronic diseases, including heart disease and diabetes, have substantially increased due to global economic changes. The most significant risk to human health comes from chronic disorders. Also, if a disease were to spread, it would cause an influx of patients to the hospitals, burdening medical facilities’ capacity to care for everyone. For instance, the global spread of COVID-19 is straining medical facilities around the globe [4]. 

Healthcare 4.0 refers to integrating digital technologies, data analytics, and AI in healthcare [1]. This includes telehealth, electronic health records, AI-driven diagnostics, and personalized medicine [5]. AI-driven diagnostics can provide accurate and timely diagnoses, improving treatment outcomes. Telehealth services can improve access to healthcare and reduce the burden on physical facilities [6]. 

The Internet of Medical Things (IoMT) is another paradigm expected to revolutionize the healthcare sector and provide novel healthcare services [7]. The IoMT is expected to continue expanding with advancements in sensor technology, connectivity, and data analytics. More medical devices will be connected, allowing for real-time monitoring, remote patient care, and early detection of health issues. It can improve patient outcomes by enabling continuous monitoring, personalized treatments, and remote care. Real-time data transmission can facilitate early intervention, reducing complications and hospital readmissions [8]. 

By leveraging technology, healthcare systems can become more proactive, personalized, and efficient. However, data security, privacy, and implementation challenges should also be addressed to maximize the benefits of these advancements [1,5].

This review work investigates the current state-of-the-art healthcare systems, including Healthcare 4.0 and IoMT. It provides a deeper understanding of Healthcare 4.0 and IoMT. The main contributions of this review are summarized as follows.

**A.**  **Providing insight into the Healthcare 4.0 paradigm and its main features.**

Researchers can benefit from understanding the Healthcare 4.0 paradigm and its main features by gaining a comprehensive understanding of the current state of healthcare technology. This knowledge can guide their research focus, helping them align their studies with the pressing needs and challenges in healthcare. Furthermore, researchers can identify gaps in the current implementation of Healthcare 4.0, offering opportunities for future research and innovation.

By having a clear understanding of the main features of Healthcare 4.0, researchers can also identify potential collaborations with industry partners and healthcare institutions. This collaboration can foster the translation of research findings into real-world applications, accelerating the adoption of Healthcare 4.0 technologies and positively impacting patient care on a broader scale.

**B.**  **Introducing the main requirement of Healthcare 4.0 systems and IoMT.**

Understanding the main requirements of Healthcare 4.0 systems empowers healthcare practitioners to adapt to the latest technological advancements in their practice. They can integrate data analytics and AI tools to make evidence-based decisions, personalize treatment plans, and improve patient outcomes. Awareness of data security and privacy requirements helps practitioners implement measures to protect medical data.

The main requirements of Healthcare 4.0 systems provide researchers with clear directions. They can focus on developing innovative technologies or solutions that address the specific needs of the healthcare industry in this paradigm. The introduction of IoMT opens up numerous research opportunities. Researchers can explore the potential of new medical devices, algorithms, and applications in remote monitoring, diagnostics, and patient care.

**C.**  **Providing the key enabling technologies of Healthcare 4.0 and IoMT.**

By providing an in-depth understanding of key enabling technologies, researchers can explore their potential and limitations, fostering innovation and discoveries in the Healthcare 4.0 paradigm. Embracing these technologies and concepts can lead to improved patient care, enhanced healthcare operations, and groundbreaking research that can shape the future of healthcare delivery. Moreover, these technologies offer researchers the tools to tackle pressing healthcare challenges and improve patient care, making their research highly relevant and impactful in shaping the future of healthcare delivery.

**D.**  **Discussing the research directions for Healthcare 4.0 and IoMT.**

Discussing research directions for Healthcare 4.0 and IoMT is essential because it helps guide researchers in identifying areas of study that are relevant, impactful, and aligned with the evolving needs of the healthcare industry. As Healthcare 4.0 continues to develop, new challenges and opportunities emerge, requiring researchers to focus their efforts on specific research directions that can bring about transformative changes in healthcare. 

Healthcare 4.0 and IoMT encompass a wide range of technologies and applications. Discussing research directions helps researchers identify specific topics that are currently relevant and have the potential to address pressing healthcare challenges. It allows researchers to prioritize their efforts on areas that can lead to significant advancements and practical solutions.

The rest of the work is organized as follows. Section 2 introduces the e-health paradigm, including definitions, challenges, and main categories. Section 3 investigates the characteristics of WBAN, distinguishing it from mobile ad hoc networks (MANETs) and wireless sensor networks (WSNs). It also introduces the categories of medical sensors and the communication interfaces used for WBANs, IoMT, and Healthcare 4.0. Section 4 presents the main features and challenges of Healthcare 4.0 and IoMT. Section 5 provides details on the key technologies that can assist the evolution of IoMT and Healthcare 4.0. Section 6 gives insights into future research directions. Section 7 presents the ethical implications of Healthcare 4.0.

## 2. E-Health

Nowadays, many people are talking about e-health, yet few of them have come up with a definition that explains this relatively new term. This term was rarely used before 1999, and now this term seems similar to a “buzzword” describing “Internet medicine” and everything related to computer-based medical services. Industry leaders and employees used this term for the first time in marketing rather than academics. They use the term proportionately to other “e-words”, e.g., e-business, e-commerce, and e-solution [9].

It described the new possibilities that the Internet opens to healthcare. For instance, Intel defines e-health as “a collaborative effort by healthcare and high technology industry leaders to realize the full potential of the Internet’s role in healthcare delivery” [10].

Recent definitions of e-health in the electronic environment place it at the point where medical informatics, public health, and commercial activities converge. It offers medical services and information via the Internet and related technologies. E-health describes not only technical aspects, but also the mindset, and way of thinking to enhance local, regional, and global healthcare using information and communication technologies (ICTs) [11].

Among the components of e-health are telemedicine, decision support systems, electronic consultations, and electronic health records (EHR). Computer-generated prescriptions have many advantages, such as linkages to programs emphasizing drug or drug groups’ hazards. Computers are now fundamental to most healthcare practices that initially give electronically recorded patient information [12].

### 2.1. Challenges of E-Health

Thanks to rapid technological breakthroughs, new e-health solutions have been continually growing to fulfill the needs of contemporary practice. Appropriate technology infrastructure, systems integration, standards, and social, ethical, and economic challenges are all major roadblocks to widespread e-health adoption and the delivery of higher-quality, more productive healthcare. In the era of modern communication networks, e-health systems are expected to meet the following demands [13,14,15].

(1) Timeliness

Healthcare systems and organizations should be structured in such a way that patients can receive care quickly in real-time. Misdiagnosis or inability to diagnose a medical problem, illness, or injury in a timely manner, while occasionally harmless, can lead to the condition worsening and worsening results. Thus, many medical services are announced as ultra-reliable low latency (URLL) services.

(2) Security and Privacy

Privacy, or the capacity to keep an individual’s medical information confidential from unauthorized parties. To achieve this goal, specific activities must be taken, such as correct patient identification, improved communication, adequate care, and reduced risk of unfavorable outcomes.

Due to the critical importance of medical data, high-security algorithms should be deployed to secure medical data. Moreover, due to the constraints on medical devices’ energy and the size of data packets communicated over the network, novel energy-aware quantum security schemes are recommended to be introduced for e-health systems.

(3) Cost efficient

Healthcare services should be reasonably priced due to their high importance. Thus, cost-efficient devices and solutions should be introduced to e-health systems to provide medical services at the expected cost.

(4) Effective care

E-health services should be supported by scientific proof, e.g., laboratory tests, clinical research, and epidemiological studies. Application servers of medical networks should have interfaces and links to medical facilities and be monitored by the operators in these facilities.

(5) Equity

It implies giving everyone the same level of care, regardless of gender, color, education, geography, or socioeconomic situation.

(6) Patient-centered healthcare

This includes tailoring healthcare services to the individual patient’s requirements, values, and preferences.

### 2.2. Main Categories of E-Health Systems

**Figure 1:**
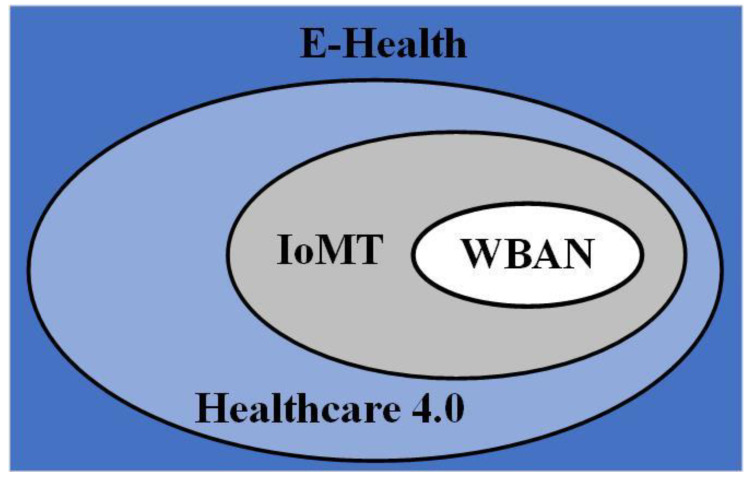
Classification of e-health systems.

The numerous ways digital and mobile technologies are used to meet health system demands are classified as e-health interventions. This classification framework, aimed primarily at public health audiences, attempts to create an accessible and bridging language for health program planners to explain the functions of e-health systems. This classification method is based on the concept of an e-health intervention, which is a distinct function of digital technology used to achieve healthcare goals [1,5]. Figure 1 provides the recent classifications of e-health systems according to technological evolution. E-health systems can be classified into wireless body area networks (WBAN), Internet of Medical Things (IoMT), and Healthcare 4.0.

WBANs typically comprise small nodes that record body health indicators, including pulse rate, blood pressure, body temperature, and glucose level. Such networks aim to provide patients with long-term health surveillance under the supervision of medical authorities without interfering with their daily lives. WBAN is a wireless network of small, low-power, and minimally invasive sensors attached to or implanted in a person’s body to monitor vital signs or other physiological data. The sensors collect and wirelessly transmit data to a central monitoring system, i.e., the sink node [16].

The IoMT is a network of medical devices and applications connected to the Internet and communicating with one another. This technology provides healthcare professionals with real-time diagnostic data and data that can be used to make more informed decisions. The IoMT consists of dense medical devices, including wearable devices, remote monitoring systems, and medical implants [8]. It works by gathering data from numerous medical devices, transmitting it to a central system or database, and analyzing it to provide insights into patient health or to enhance healthcare workflows. IoMT is viewed as a tool to enhance patient outcomes, lower healthcare expenses, and boost the effectiveness of healthcare delivery [17].

WBAN focuses specifically on monitoring physiological parameters and collecting data directly from the human body. However, IoMT involves a broader ecosystem of medical devices and technologies beyond the body itself [18].

Healthcare 4.0 or Health 4.0 is the term used to describe the fourth industrial revolution in healthcare, involving integrating advanced digital technologies, e.g., AI, and IoT devices to enhance healthcare delivery systems [19]. It is an advanced form of healthcare that incorporates digital and networking technologies, leading to enhanced patient care.

Healthcare 4.0 is the next evolution of the healthcare industry that has the potential to reshape the way healthcare is delivered, making it more efficient, effective, and accessible to everyone. Healthcare 4.0 goes beyond just technology and focuses on transforming the entire healthcare delivery system. It encompasses IoMT as a part of its larger framework [20].

## 3. Wireless Body Area Network (WBAN)

### 3.1. Characteristics of WBAN

This section investigates the characteristics of WBAN, distinguishing it from MANETs and WSNs. WBANs are interconnected devices mounted on, in, or around the human body. Medically implanted and wearable sensors are wildly popular in the context of E-Healthcare. IEEE 802.15.6 is an alternative name for the WBAN standard created by the IEEE 802.15 working group [21]. The structure of a WBAN typically includes the following components [16,22]:

(1) Medical sensors: These small, low-power devices collect data from the human body. They can be attached to the skin, implanted in tissues, or ingested by the patient.
(2) Wireless transceivers: The hardware responsible for sending and receiving data between the sensors and the central monitoring system. Various wireless communication technologies, including Bluetooth, Zigbee, or Wi-Fi, can be supported by the WBAN transceivers.
(3) Central monitoring system: The network sink, i.e., the coordinator device receives and processes the data from the distributed medical sensors. It has higher communication capabilities and supports multiple communication interfaces. A body control unit (BCU), a body gateway, or a sink are all terms used to describe this equipment. A personal digital assistant (PDA) or smartphone can sometimes be used instead of a dedicated unit. The primary goal of this unit is to collect all of the data collected by the sensors and actuators and convey it to the user (patient, nurse, etc.) over an external gateway. This gadget has a power unit, a huge processor, vast memory, and a transmitter.

Several important distinct characteristics distinguish WBAN, summarized in Table 1 [23,24,25].

Numerous benefits of WBANs make them more appealing to researchers and the industry compared to traditional medical strategies and old sensor networks [22]. They are non-surgical networks that autonomously monitor and transmit the patient’s health status to a nearby coordinator device. Then, this device transmits medical information to hospital-based healthcare professionals for further analysis to make the appropriate medical decision for the patient. Such monitoring saves time and money because it permits early detection and intervention of health issues without violating patient privacy or employing full-time medical personnel [16].

WBANs have many applications; however, most are related to simple and traditional healthcare monitoring [26]. The structure of a WBAN is designed to enable continuous and noninvasive monitoring of a patient’s health status. This can be particularly useful for people with chronic medical conditions or those requiring close monitoring during surgery recovery [27]. Few recent applications have seen the development and deployment of WBANs to improve the lives of people with mental and physical disabilities, specific diseases, and pregnant women. The main categories of WBAN are summarized as follows [25,28].

- General treatment and diagnosis application: WBAN has dominated the medical industry by providing diverse services. It improves the efficiency of medical activities, including remote patient monitoring, prompt health status, notification, and emergency phoning, which can be carried out anytime and from any location. Some of the research’s potential medical applications are listed below.
- Electronic healthcare monitoring for older people: This WBAN application aims to improve the health of older people who live alone in their homes. An intelligent home monitoring system based on medical sensors can be used to observe and evaluate the fitness of older people in their homes. Temperature sensors and other body sensors are used to detect any abnormalities in the daily activities of older people, including sleeping, walking, eating, bathing, and even operating. The network coordinator is notified if an irregularity occurs, and the collected data is sent to medical personnel.
- Fighting COVID-19: The WBAN has many applications for fighting COVID-19. By reducing the physical presence of patients, remote monitoring of COVID-19 may significantly lower healthcare costs and boost hospital capacity. Biomedical parameters such as temperature, heartbeat, respiration rate, and oxygen saturation must be collected, analyzed, and forwarded to medical personnel to monitor the symptoms [28]. WBAN can be easily deployed to assist such applications.

### 3.2. Available Medical Sensors

Healthcare 4.0 requires integrating new and emerging technologies, including IoMT and WBANs, into healthcare systems. It is an era of big data systems comprising massive data from various sources. These data types are summarized as follows [29,30].

(a) Patient data: This includes personal information of the patient, their medical history, vital signs, and any other relevant information related to their health.
(b) Clinical data: This includes data from medical devices, vital signs monitoring systems, and other clinical instruments used in patient care.
(c) Electronic health records (EHRs): EHRs store digital versions of a patient’s medical record, which includes information about their medical history, laboratory test results, and any other information related to their health.
(d) Wearable data: Wearable devices, such as smartwatches, fitness trackers, and health monitors, provide data on a patient’s activity levels, sleep patterns, and overall health.
(e) Social determinants of health: This includes factors such as a patient’s socioeconomic status, living conditions, and lifestyle choices that can impact their health.

These data are generated from various sources. This section introduces the main available wireless medical sensors deployed for WBANs, IoMT, and Healthcare 4.0. A plurality of available medical sensors is used to monitor patients’ vitals in real-time and measure a wide range of biological variables [31]. These sensors can be categorized as follows [31,32,33].

1. Wearable health trackers include activity, heart rate, and blood pressure monitors. Table 2 presents the features of the common market available wearable watches.
2. Connected health sensors and monitors include blood glucose meters, pulse oximeters, and electrocardiography (ECG). These sensors enable real-time monitoring of medical conditions, such as cardiac disease, diabetes, and respiratory disease.
3. Smart pill sensors monitor medication adherence and provide feedback to healthcare providers.
4. Telehealth and virtual care devices that enable remote consultations, diagnosis, and treatment of patients.
5. Endoscopic pills are small, swallowable devices used in medical diagnosing.

In the rest of this section, we introduce the most common medical sensors.

**A.**  **Sensors for pulse rate**

Pulse rate sensors, also known as heart rate sensors, are devices used to measure an individual’s heart rate or pulse rate. They are commonly used in fitness trackers, smartwatches, and medical devices. There are many pulse rate sensors; however, photoplethysmography (PPG) is a common technique. PPG sensors detect changes in blood volume using light. Its operation mechanism depends on emitting light, usually green or red, into the user’s skin or fingertip. Blood vessels in the skin or fingertip absorb some of the emitted light while the remaining light is reflected to the sensor [40]. As the heart beats, blood flow changes, causing variations in the absorption and reflection of light. The sensor detects these changes. The detected light variations are converted into electrical signals and then processed to determine the heart rate. The processed data can be displayed on a screen or transmitted to a connected device for further analysis or tracking of the user’s pulse rate [41].

It Is significant to mention that various pulse rate sensors may vary on this basic method. Some more advanced sensors employ additional technologies, such as the electrocardiogram (ECG), for more precise measurements. The different types of market-available heart rate sensors are introduced as follows [40,41,42].

(1) Optical sensor: It uses light to detect changes in blood volume. A light-emitting diode (LED) and a photodetector measure the reflected light. When blood flow increases during each heartbeat, the amount of light reflected in the sensor changes, allowing the pulse rate to be calculated.
(2) Piezoelectric sensor: It uses piezoelectric materials that generate voltage when subjected to mechanical pressure or vibration. In the context of pulse rate measurement, a piezoelectric sensor can be placed on the skin or a blood vessel, and the mechanical vibrations caused by each heartbeat can be converted into an electrical signal to determine the pulse rate.
(3) Capacitive sensor: It measures changes in capacitance between two electrodes caused by the pulsating blood flow. These sensors typically involve placing the electrodes on the skin or blood vessels, and as the blood flow varies with each heartbeat, the capacitance also changes.
(4) ECG sensor: It can provide information about the heart’s electrical activity. ECG sensor detects the electrical signals generated by the heart’s contractions and relaxations. Various parameters, including heart rate, can be derived from these signals.

Selecting pulse rate sensors, for different medical applications, should consider several factors that are summarized in Table 3 [40,41,42].

**B.**  **Pulse oximeters**

A pulse oximeter is a medical device used to measure the oxygen saturation level (SpO2) in a person’s blood. It is a small, portable device that typically clips onto a finger, toe, or earlobe. The pulse oximeter emits two wavelengths of light, typically red and infrared, into the body part to which it is affixed. The device then measures the light absorbed by the blood’s oxygen-carrying hemoglobin. Based on this measurement, the oxygen saturation level is calculated and expressed as a percentage. Pulse oximeters are commonly utilized in hospitals and clinics but are also available at home. They are particularly useful for monitoring patients with respiratory or cardiac conditions and determining the general health status of individuals during physical activity or at high altitudes [43].

A blood oximeter can detect asthma, pneumonia, anemia, lung disorders, and other medical conditions. Although a blood oximeter reading of peripheral SpO2 is not always comparable to the preferred arterial SpO2, it is considered safe, convenient, noninvasive, and affordable. The pulse oximetry method is a valuable tool for assessing oxygen saturation in clinical practice [44].

Pulse oximeters work based on the principle of spectrophotometry. They emit two beams of light, typically red and infrared, through the body part where it is attached. Oxygenated hemoglobin (carrying oxygen) absorbs more infrared light, while deoxygenated hemoglobin (without oxygen) absorbs more red light. The pulse oximeter measures how much light the blood absorbs and calculates the oxygen saturation level by comparing the absorbed red and infrared light ratio. This data is then displayed on the device’s screen, showing the oxygen saturation percentage (SpO2) and the pulse rate. The output of a pulse oximeter indicates the efficiency with which oxygen is transported to various body regions. Normal oxygen saturation levels range from 95 to 100 percent. Hypoxemia, characterized by insufficient oxygen levels in the blood, may be indicated by values below this range [45].

A common use case of pulse oximeters is their use in airplanes [46]. Airplanes need such a system because passengers with chronic obstructive pulmonary disease (COPD) are at risk from the low pressure, low oxygen levels, and low humidity that can occur within the cabin during the flight. Therefore, it is essential to monitor their health and recognize any irregularities promptly so that they can receive appropriate care.

**C.**  **PPG sensors**

A photoplethysmography (PPG) sensor is a device that measures changes in peripheral blood vessel blood volume. It detects variations in the absorption or reflection of light caused by blood flow using light sensors. PPG sensors are commonly found in wearable devices such as fitness trackers and smartwatches to estimate blood oxygen levels and monitor pulse rates. The sensor emits light onto the epidermis and measures the intensity of the light that is reflected, producing a photoplethysmography waveform. This waveform can provide insight into various health parameters and is frequently employed in applications related to healthcare and wellness [47].

PPG is a common optical monitoring method used to track heart rate at a low cost. PPG does not require any incisions. A PPG sensor measures changes in blood flow by placing a light source and a photodetector on the skin’s surface. The PPG signal has recently attracted the attention of several academics interested in extracting further data from it [48]. The PPG signal’s second derivative wave carries crucial diagnostic information. Therefore, this waveform analysis can aid in identifying many cardiovascular problems. Placement of the PPG sensor on the patient’s finger, earlobe, or forehead is the norm. Researchers are exploring several anatomical zones in search of more convenient measurement points.

**D.**  **Temperature sensors**

A temperature sensor is a common medical sensor used to measure body temperature accurately and safely. It is designed specifically for medical applications to monitor patients’ temperature in hospitals, clinics, and other healthcare settings. These sensors utilize various technologies such as infrared, thermocouples, or thermistors to detect and convert body heat into an electrical signal that healthcare professionals can measure and interpret [49].

Medical temperature sensors come in different forms, including oral, rectal, ear, and forehead, and wearable patches or bands. Each type has its own advantages and limitations, and its usage depends on the patient’s age, condition, and the healthcare provider’s preference.

These sensors are crucial in detecting fever, a common sign of many illnesses. Monitoring body temperature, accurately, helps healthcare professionals diagnose and monitor the progress of diseases or infections. It enables timely intervention and appropriate treatment, ensuring the well-being of patients [50].

**E.**  **Blood pressure sensors**

Blood pressure sensors measure and monitor blood pressure levels by detecting the pressure exerted by blood flow in the arteries. Modern blood pressure sensors often employ an oscillometer, which measures the pressure changes caused by the pulsatile flow of blood. The sensor inflates the cuff to restrict blood flow temporarily and then gradually deflates it while monitoring the pressure changes. The sensor can determine systolic and diastolic blood pressure values based on these measurements [51].

Various types of blood pressure sensors are available, including those designed for home use, clinical settings, and wearable devices. Some sensors may connect to smartphones or other devices for data storage and analysis. It is always a good idea to consult a healthcare professional for advice on selecting and using blood pressure sensors, as accurate measurements and proper technique are essential for monitoring and managing blood pressure effectively. Alternative methods use biological sensors such as ECG and PPG sensors to estimate blood pressure without a cuff [52].

**F.**  **EEG sensors**

Electroencephalography (EEG) sensors are devices used to detect and record the electrical activity of the brain. EEG is a non-invasive neuroimaging technique that measures the voltage fluctuations resulting from the ionic current flows within the neurons of the brain. These sensors have a wide range of applications, including diagnosing epilepsy, sleep disorders, brain injuries, and neurological conditions. They are also used in neurofeedback, brain-computer interfaces (BCI), and cognitive research [53].

EEG sensors consist of multiple electrodes that are typically attached to the scalp. These electrodes are small metal discs or sensors that come into contact with the scalp to pick up the electrical signals produced by the brain. Electrodes are either wet or dry. Wet electrodes require a conductive gel to be applied between the electrode and the scalp to ensure good electrical contact. The gel helps to reduce impedance and interference. While dry electrodes do not require any additional gel and can be directly applied to the scalp [54]. They are more user-friendly and are commonly used in consumer-grade EEG devices. The number of electrodes used in an EEG system can vary, ranging from a few channels to several hundred, depending on the level of spatial resolution required. The positioning of EEG electrodes is essential to capture specific brain activity patterns accurately [55]. 

Moreover, EEG sensors are connected to amplifiers that strengthen the weak electrical signals picked up by the electrodes. Amplifiers play a crucial role in maintaining the signal-to-noise ratio, which is critical in obtaining high-quality EEG data. The amplified analog signals are then converted to digital signals using analog-to-digital converters (ADCs). Digital signals are easier to process, store, and transmit for further analysis.

The EEG system includes a signal processing unit that filters, amplifies, and digitizes the raw EEG data. Signal processing techniques are employed to remove artifacts and noise from the signals. Modern EEG systems often have data interfaces, e.g., Bluetooth, allowing real-time data streaming and integration with other devices or software applications [56].

**G.**  **EMG sensors**

Electromyography (EMG) sensors detect the electrical activity produced by muscles. These devices are commonly used in medical and research fields to assess muscle function, diagnose neuromuscular disorders, and control prosthetic limbs. EMG sensors consist of electrodes placed on the skin surface above the muscles of interest. These electrodes pick up the electrical signals generated by muscle contractions. When you move a muscle, electrical impulses are sent from your brain through your nerves to the targeted muscle fibers, causing them to contract. The EMG sensor detects these electrical signals and converts them into measurable readings [57].

There are two types of EMG sensors: surface EMG and intramuscular EMG. Surface EMG sensors use adhesive electrodes placed on the skin, while intramuscular EMG sensors involve inserting fine needle electrodes directly into the muscle [58]. EMG sensors are valuable in various applications, including medical diagnostics, sports science, rehabilitation, and human-computer interfaces. They enable researchers and clinicians to assess muscle function, monitor progress during therapy, and develop innovative technologies that interface with the human body [59].

Medical professionals use EMG sensors to keep track of a patient’s nerve and muscle problems. Wearable gadgets that monitor a patient’s behavior use such sensors as well. In systems that use emotion-based intelligent information sensing, EMG sensors prove to be essential. There are systems that use EMG signals and other body vital monitoring biomedical sensors to evaluate face muscle variability and classify each change with its related emotion [60]. Table 4 presents the common market available IoT medical sensors. Also, the table introduces the main specifications of each sensor [61].

### 3.3. Wireless Communication Technologies

With the recent evolution of wireless technology, several wireless communication technologies have been introduced to wireless medical sensors used in Healthcare 4.0 and IoMT. These heterogeneous interfaces are commonly used to enable connectivity and data exchange. The key wireless technologies deployed in medical IoT applications are introduced as follows [71,72,73,74].

- Bluetooth: Bluetooth technology is widely adopted for connecting medical devices and wearables to smartphones, tablets, and other computing devices. It allows for the transmission of health data, remote device control, and seamless integration with mobile health applications.
- Bluetooth Low Energy (BLE): It is a wireless communication technology designed to provide short-range, low-power connectivity between devices. BLE is a subset of the Bluetooth technology standard and is optimized for applications that require low power consumption and periodic data exchanges. BLE is specifically designed for energy-efficient communication, making it ideal for devices that operate on battery power. This allows BLE-enabled devices to have long battery life, which is essential for applications such as wearables, medical devices, and IoT sensors. BLE provides short-range communication typically within a range of up to 10 m. This limited range is well-suited for medical applications that require local and proximity-based interactions between devices. Compared to classic Bluetooth, BLE has a lower data transfer rate. It is suitable for transmitting small bursts of data, such as sensor readings, notifications, or control commands.
- Zigbee: Zigbee is a low-power, short-range wireless technology well-suited for medical IoT applications. It is an IEEE 802.15.4-based. Due to its energy efficiency and reliable connectivity, it is often used in remote patient monitoring systems, smart home healthcare devices, and hospital equipment monitoring systems.
- Wi-Fi: Wi-Fi is a popular wireless method for establishing a direct internet connection. It utilizes the 2.4 GHz frequency spectrum frequently. The family of IEEE 802.11x standards is referred to as “Wi-Fi.” It achieves a theoretical coverage of 20 to 100 m indoors. Also, it allows for a maximal transfer rate of more than 54 Mbps. In numerous ways, it outperforms many existing communication interfaces. Wi-Fi fits well for audio and video applications due to its higher bandwidth requirements. Deploying Wi-Fi for IoT applications consumes a lot of energy, which is one of its primary disadvantages. It is not feasible for IoT sensors to run on batteries because of their high power consumption. Additionally, it is especially vulnerable to background interference and channel obstruction. Wi-Fi is, without a doubt, essential for high-speed connections. However, it has significant limitations and disadvantages in the context of the IoT, rendering it less popular. Nonetheless, many researchers have proposed healthcare systems incorporating Wi-Fi in monitoring patients’ vitals. Wi-Fi provides high-speed wireless connectivity over short to medium distances. It is extensively used in healthcare settings for connecting medical devices, including wearable health trackers, patient monitoring systems, and smart hospital infrastructure.
- Cellular technology: Cellular networks provide wide-area coverage and are utilized for medical IoT applications that require long-range communication, such as telemedicine and remote patient monitoring. The evolution of cellular networks from 3G to 5G facilitates higher data rates, lower latency, and improved connectivity.
- LoRaWAN: LoRaWAN (Long Range Wide Area Network) is a low-power, long-range wireless technology suitable for medical IoT deployments that span large areas, such as smart hospitals or smart cities. It enables connectivity with low-cost, battery-operated devices and supports efficient data transmission in scenarios where power consumption is critical.
- 6LoWPAN: It is the IPv6 transmission over a low-power wireless personal area network. It is cheap, energy efficient, and easy to tailor to your needs. With these features, it can be used for various IoT tasks. It supports IPv6 and a variety of IEEE 802.15.4 protocols for data exchange.

## 4. Mian Features and Challenges of IoMT and Healthcare 4.0 

### 4.1. Specifications and Requirements of IoMT and Healthcare 4.0

Healthcare 4.0 aims to improve patient outcomes and access to healthcare, increase efficiency, and reduce costs. It includes using EHRs, telemedicine, wearable devices, health information exchanges, big data analytics, and machine learning to provide personalized, preventive, and precise healthcare [1]. This promising paradigm, with IoMT, has numerous benefits for patients and healthcare workers, summarized as follows [17,75,76].

1. Better healthcare services: IoMT devices can monitor patients’ health and vital signs, enabling healthcare providers to detect and address prospective problems. These processes are provided over a robust system since IoT has evolved in the past years.
2. Cost-efficient: By reducing hospital readmissions, minimizing unnecessary interventions, and optimizing the utilization of medical resources, IoMT devices can help reduce the cost of healthcare. The infrastructure of IoMT is cost-efficient, and the system does not involve medical workers all over time since the system automatically analyzes the results.
3. Enhanced patient engagement: IoMT devices can increase patient engagement in healthcare by allowing patients to monitor their health, assess their progress, and communicate with their healthcare providers.
4. Improved decision-making: Healthcare 4.0 can provide healthcare providers with real-time data and insights, allowing them to make more informed decisions and provide patients with more personalized care.
5. Better interaction efficiency: Healthcare 4.0 can assist healthcare providers in streamlining their workflows, enhancing communication, and increasing the efficiency of patient care.

This new era of telemedicine has many requirements that can be summarized as follows [5,77,78].

1. Advanced data analytics: The advanced data analytics capability is crucial to Healthcare 4.0. It enables the identification of anomalies and patterns that can assist in providing enhanced personalized care, thereby leading to better patient outcomes.
2. Ultra-reliable communication: It is a critical aspect of medical data transferred over Healthcare 4.0, enabling dependable, safe, and operational communication among healthcare actors. It ensures that data is communicated without any loss or breakdowns, providing reliable diagnosis, treatment decisions to physicians, patients, and caregivers. Ultra-reliable communication ensures that critical medical data, including vital signs, lab results, and medication information, are transmitted accurately and rapidly to the right recipients. Moreover, it can enable timely communication of alarm notifications, emergency calls, and other urgent messages, which can help prevent medical errors and reduce the risk of adverse events. Summing up, ultra-reliable communications are crucial in improving patient safety and clinical outcomes by enabling reliable and real-time communication between healthcare providers, patients, and medical devices.
3. High network availability: The high network availability of Healthcare 4.0 data refers to the healthcare system’s ability to access and utilize data in real-time and without interruption. Having a highly available network ensures that healthcare providers always have access to patient records, medical images, and other vital data, which can save lives in critical situations.
4. Interoperability: Interoperability is essential in the Healthcare 4.0 environment. This necessitates that all systems and devices communicate with one another, allowing for the sharing and analysis of data. Healthcare 4.0 systems significantly rely on the ability to share patient data across systems.
5. Information consent: Patients should have control over their health data and how it is disseminated, per informed consent. Before using patients’ data, healthcare organizations must ensure that patients are completely informed about how it will be utilized and obtain their consent.
6. High network flexibility: Network flexibility is a key component of Healthcare 4.0, which indicates the ability of healthcare providers to adapt their networks quickly and easily to changing patient needs and technological advancements. This is particularly important as Healthcare 4.0 involves the integration of various technologies, including robotics, AI, the IoT, and big data analytics. The ability to add these technologies quickly and seamlessly to the healthcare network is essential in order to improve patient care, productivity, and overall healthcare quality.
7. Security and privacy: Most countries store and transmit personal health information (PHI) electronically. Due to the vast amount of health information gathered and shared via the Internet, the key concerns of healthcare operators are privacy and security. There is a risk of system-wide attacks on health data due to the open transmission channel. To defend against such attacks, researchers employ a variety of cryptographic strategies. Privacy includes the ability of only authorized users to access health information about specific patients, as well as the retrieval, use, and disclosure of patient information to an interloper. This can be achieved by implementing various schemes and their corresponding rules.

### 4.2. Challenges with IoMT and Healthcare 4.0

Developing communication networks for Healthcare 4.0 and IoMT faces many challenges. In this section, we address these limitations.

**A.**  **Data privacy and security**

The increased reliance on EHRs, connected devices, and health applications creates a higher risk of data breaches and unauthorized access to sensitive patient information. Data privacy and security in Healthcare 4.0 are critical considerations due to the increasing reliance on technology, data, and interconnected systems. Some of the Healthcare 4.0 privacy and security challenges are introduced as follows [79,80].

- Data breaches: The digitization and sharing of vast amounts of patient data increases the risk of data breaches. Cybercriminals may attempt to gain unauthorized access to sensitive health information, leading to identity theft, fraud, or other malicious activities.
- Internal threats: Healthcare organizations must also be wary of internal threats. Employees with access to patient data may intentionally or unintentionally misuse or disclose confidential information.
- Interoperability risks: Integrating various systems, devices, and applications can create vulnerabilities in data exchange, leaving data exposed during transmission or storage. Ensuring secure communication and data sharing among different platforms is crucial.
- IoT vulnerabilities: The growing use of IoMT devices in healthcare presents security challenges. Weaknesses in these devices can be exploited, compromising patient data and device functionality.
- Lack of standardization: Inconsistencies in security practices and protocols across different healthcare organizations can lead to weaknesses that attackers may exploit. The lack of standardized security measures can hinder overall system security.
- Ransomware attacks: Healthcare facilities have increasingly become targets of ransomware attacks where hackers encrypt patient data, making it inaccessible until a ransom is paid. Such attacks can disrupt patient care and compromise data integrity.
- Inadequate security measures: Some healthcare providers may not implement robust security measures due to cost constraints or lack of awareness. Insufficient security measures can expose data to potential breaches.
- Data sharing with third parties: Healthcare providers often share patient data with third-party vendors and partners for various purposes. Ensuring the security of data during such transfers is crucial to prevent data leaks.

Addressing these challenges requires a comprehensive and multi-layered approach to data privacy and security. This includes [79,80,81]:

- Implementing robust cybersecurity measures, encryption, access controls, and intrusion detection methods.
- Conducting regular security audits and risk assessments to identify and mitigate vulnerabilities.
- Educating employees about data security best practices and raising awareness of potential threats.
- Adopting standardized security protocols and collaborating with industry peers to share best practices.
- Employing secure application development practices for software used in healthcare.
- Regularly updating and patching software and systems to address known security vulnerabilities.
- Restricting data access to authorized personnel only and monitoring access logs for suspicious activities.
- Establishing clear data-sharing agreements with third-party vendors to ensure data protection compliance.

**B.**  **Bandwidth**

Bandwidth challenges in Healthcare 4.0 systems arise due to the growing demand for data-intensive applications and the proliferation of connected devices and technologies in the healthcare industry. Also, the 5G demands for medical applications introduce many issues with the bandwidth [77].

Healthcare 4.0 systems involve the use of data-intensive applications such as telemedicine, medical imaging, and real-time patient monitoring. These applications generate and transfer large volumes of data, putting a strain on the available network bandwidth. Moreover, With the rise of telemedicine, video conferencing has become a critical aspect of healthcare communication. High-quality video and audio streams necessitate sufficient bandwidth for smooth and uninterrupted interactions [20].

Addressing bandwidth challenges in Healthcare 4.0 systems can be achieved using the following approaches [20,77]:

- Upgrading network infrastructure: Healthcare providers need to invest in high-speed and reliable network infrastructure to accommodate the increasing demands of data-intensive applications.
- Prioritizing critical data traffic: Implementing quality of service (QoS) protocols can help prioritize critical healthcare data through time-sensitive traffic, ensuring that vital patient information receives priority during peak periods.
- Edge computing: Deploying edge computing solutions can reduce the strain on centralized networks by processing data closer to the source, thereby decreasing the volume of data that needs to be transferred over the network.
- Data compression: Implementing data compression techniques can help reduce data size, easing the burden on the network and improving efficiency.
- Load balancing: Load balancing techniques distribute network traffic across multiple servers or resources, preventing congestion and ensuring even data distribution.
- Bandwidth monitoring and management: Continuous monitoring of network bandwidth usage helps identify bottlenecks and allows for proactive capacity planning and management. Also, applying AI-based methods at the core of the network can assist bandwidth management.

**C.**  **Energy**

Energy in healthcare systems and devices encompasses several issues related to power supply, energy efficiency, and sustainability. These challenges are critical in Healthcare 4.0, which relies heavily on energy-limited devices, i.e., IoMT. Table 5 presents the key energy challenges with Healthcare 4.0 [5,18].

Some potential solutions to overcome such challenges include the following [5,18]:

- Energy-efficient medical devices: Healthcare organizations should prioritize the adoption of energy-efficient medical devices and technology. Manufacturers should design products with power-saving features and low standby power consumption.
- Battery technology improvements: Ongoing research and development in battery technology can lead to longer-lasting and more energy-dense batteries for medical devices and mobile equipment.
- Energy management techniques: Implementing energy management methods can help optimize energy use, track consumption, and ensure reliable power supply during emergencies.
- Renewable energy adoption: Investigating novel methods for the integration of renewable energy sources. Also, developing robust models and systems for wireless charging can assist the integration of green energy in Healthcare 4.0.

**D.**  **Massive data**

Healthcare 4.0 presents significant challenges related to handling massive amounts of data. Table 6 introduces these challenges [82,83].

**E.**  **Ethical concerns**

The ethical concerns of Healthcare 4.0 are introduced in detail in Section 7.

Summing up, Table 7 summarizes the challenges and limitations of Healthcare 4.0 and introduces the potential key solutions for such limitations.

## 5. Key Enabling Technologies of Healthcare 4.0

**Figure 2:**
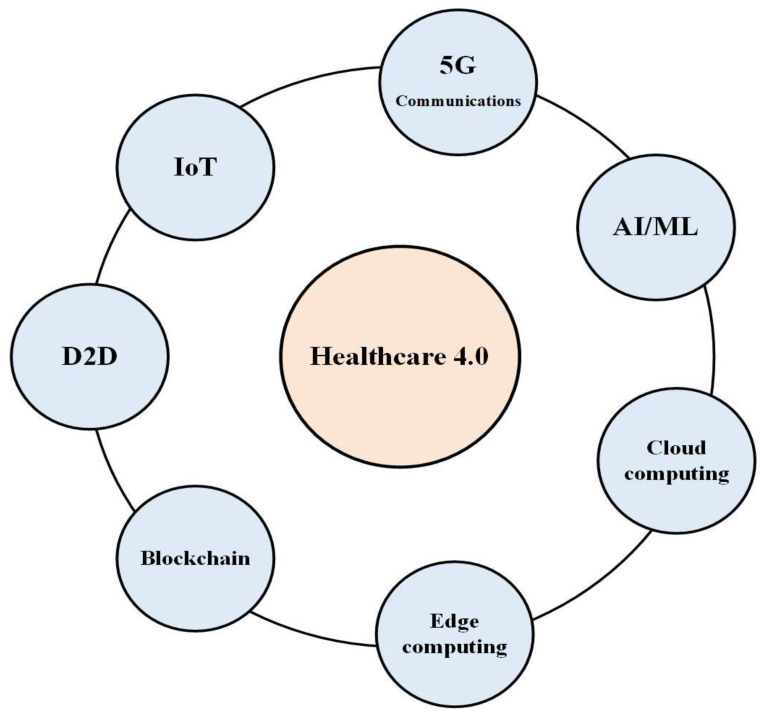
Key enabling technologies of Healthcare 4.0.

To meet the demands of Healthcare 4.0, novel technologies should be integrated via a reliable network structure. Figure 2 presents the main key enabling technologies of Healthcare 4.0 [20].

Wearables and IoT devices have gained significance in the Healthcare 4.0 ecosystem. IoT devices can capture real-time patient data, monitor medical equipment, and track inventory, enhancing patient outcomes and decreasing costs [29]. It provides efficient technology due to the high flexibility supported by IoT networks. Moreover, the availability and plurality of medical sensors facilitate the introduction of IoT in Healthcare 4.0 systems. A detailed analysis of medical IoT devices that can be used to assist Healthcare 4.0 was introduced in Section 3.2.

### 5.1. 5G Communications

The 5G communications can play critical roles in Healthcare 4.0 systems by providing an ultra-reliable low-latency communication medium [84]. It can greatly assist Healthcare 4.0 by enabling advanced connectivity and technology integration within the healthcare industry. This efficiently sets up many applications, including telesurgery operations and remote critical diagnosis. The 5G communications can empower Healthcare 4.0 in several ways that are summarized in Table 8 [84,85].

To better introduce the benefits of 5G communications to Healthcare 4.0, we consider the following use case [85,86,87]:

- Telemedicine services: 5G can revolutionize telemedicine by enabling high-quality video conferencing and real-time collaboration between healthcare professionals. The low latency and stable connections can offer a more immersive and interactive virtual healthcare experience, leading to better diagnosis, treatment, and follow-up care.
- Remote surgeries and robotic-assisted operations: 5G’s ultra-reliable and low-latency communications can enable remote surgeries and robotic-assisted procedures. Surgeons can perform complex operations from a remote location using high-resolution video feeds and haptic feedback. This can bridge the gap between medical expertise and underserved areas, where access to specialized healthcare is limited.
- Precision medicine services: 5G empowers precision medicine initiatives by enabling fast and reliable transmission of large genomic datasets, facilitating quicker analysis and personalized treatment plans. It supports real-time collaboration among researchers and clinicians for faster advancements in precision medicine.
- Augmented and virtual reality (AR/VR) services: 5G can power immersive AR/VR applications in healthcare. This technology can be used for medical training, patient education, pain management, and even remote collaborative surgeries, fostering better outcomes and experiences for both patients and healthcare professionals.

The 5G communications have the potential to significantly improve Healthcare 4.0; however, there are several challenges and limitations to implementing 5G communications in Healthcare 4.0. systems. These limitations are summarized in Table 9 [84,85].

### 5.2. AI

AI plays a critical role as a key enabling technology in the context of Healthcare 4.0 since it can revolutionize various aspects of healthcare. For medical imaging, AI-powered algorithms can analyze medical images, such as X-rays, computerized tomography (CT) scans, and magnetic resonance imaging (MRI), to detect abnormalities and assist radiologists in making accurate diagnoses [88].

AI systems can analyze vast amounts of patient data, including medical records, symptoms, and genetic information, to provide faster and more accurate disease diagnoses, improving patient outcomes. Drug discovery is another field in which AI can assist. AI algorithms can rapidly analyze large databases of pharmaceutical compounds and predict their efficacy, helping researchers identify potential drug candidates faster and reduce the time required for clinical trials.

AI algorithms can also assist personalized medicine services by analyzing genetic data and patient records to develop personalized treatment plans, considering individual variations, and optimizing healthcare interventions. Moreover, AI can power remote monitoring services by allowing remote healthcare providers to detect changes or anomalies and provide timely interventions. AI chatbots and virtual assistants can provide basic healthcare information, answer patient queries, and offer support, enhancing patient engagement and accessibility to healthcare services. Another important aspect is the disease prediction that AI solutions can accomplish [89].

Telesurgery is another domain that has to make use of AI. To achieve the required latency and quality of experience (QoE) required by telesurgery applications. AI will be used to build model mediation algorithms and edge intelligence computing to enable real-time communication required for telesurgery applications [90].

The integration of AI in Healthcare 4.0 has the potential to transform healthcare delivery, enhance diagnostics, and improve patient care by leveraging the power of machine learning, natural language processing, and advanced analytics.

### 5.3. Cloud Computing

Cloud computing provides the storage, management, and processing of healthcare data and applications on remote servers accessed via the Internet. It offers various benefits for medical professionals and the healthcare industry. Cloud computing in healthcare promotes efficient data management, collaboration, remote access, and cost optimization for medical professionals, thereby improving patient care and streamlining healthcare processes. It achieves various benefits and functionalities to Healthcare 4.0 and IoMT, summarized in Table 10 [91,92].

### 5.4. Distributed Edge Computing

Distributed edge computing is a decentralized computing infrastructure that brings computational power and data storage closer to the edge of a network, typically near the source of data generation. It aims to reduce latency, enhance real-time processing, improve bandwidth utilization, and enhance security and privacy. The utilization of computing resources locally at the edge of the network, e.g., near the medical devices, to process and analyze data generated by IoMT devices plays a crucial role in optimizing healthcare systems [93]. 

This approach offers several benefits, including reduced latency, improved reliability, and enhanced data privacy and security. By deploying edge computing in Healthcare 4.0 and IoMT, medical data can be processed locally, minimizing the need for sending sensitive patient information to a central location. Edge nodes can perform real-time data analytics, support remote monitoring, facilitate faster response times, and enable autonomous medical device operations. However, specific deployment strategies and implementations vary across different companies and organizations. Factors such as network architecture, device capabilities, data processing requirements, and regulatory considerations influence how distributed edge computing is implemented in the IoMT [94].

Deploying distributed edge computing, e.g., fog computing and mobile edge computing (MEC), for Healthcare 4.0 and IoMT can introduce the benefits introduced in Table 11 [93,94,95].

### 5.5. Blockchain

Blockchain is a promising technology that can assist Healthcare 4.0 and IoMT applications in various ways. It can offer robust solutions to address data security, interoperability, transparency, and patient control challenges. These are critical issues with Healthcare 4.0 and IoMT systems [96]. This ultimately improves the efficacy and reliability of healthcare systems. Blockchain can assist Healthcare 4.0 and IoMT systems as follows [96,97,98].

1. Data security and integrity: Blockchain provides a transparent and immutable ledger where data transactions can be securely recorded. It utilizes cryptographic algorithms to create a tamper-proof and transparent record of all transactions or changes made to the data. Once a block is added to the chain, it cannot be altered without consensus among the network participants. This ensures that patient data remains unchanged and maintains its integrity. Blockchain ensures the integrity and authenticity of medical data IoMT devices generate, reducing the risk of tampering or unauthorized access. This strengthens data security and privacy, which is critical in healthcare.
2. Interoperability and standardization: IoMT devices often come from different manufacturers and use various data formats and protocols. A decentralized and standardized framework can be established with blockchain, allowing seamless data exchange and interoperability between different IoMT devices. This promotes collaboration, efficiency, and better patient care.
3. Decentralization: By leveraging distributed ledger technology, blockchain eliminates the need for a central authority to govern data transactions. This can enhance trust among stakeholders within the IoMT ecosystem, such as patients, healthcare providers, insurers, and regulators. The decentralized nature of blockchain ensures that no single entity controls the stored data, reducing the potential for abuse or manipulation.
4. Data control and consent management: Patients can have greater control over their health data through blockchain. They can grant or revoke access permissions to their medical records, ensuring data privacy and enabling consent-based data sharing. This empowers patients to have a say in how their data is used, fostering trust and transparency.
5. Data sharing: Smart contracts are self-executing agreements embedded in the blockchain that can be used to define access rights and data-sharing permissions. These contracts can automate consent management, ensuring that data is only shared with authorized entities or for specific purposes. It eliminates the need for intermediaries and strengthens data privacy.
6. Patient-centricity: Patients can have greater control over their healthcare information through blockchain. Patients can grant specific healthcare providers or researchers permission to access their data, enhancing patient privacy and consent management.
7. Supply chain integrity: In the healthcare industry, blockchain can improve the traceability and transparency of medical devices, pharmaceuticals, and supplies. It enables the verification of the entire supply chain journey, ensuring authenticity, reducing counterfeit products, and improving patient safety.

However, deploying a blockchain in Healthcare 4.0 and IoMT faces many challenges. Some of these include the scalability of the blockchain network to handle a massive amount of medical device data, ensuring data privacy compliance, addressing regulatory concerns, and establishing standardized protocols for data exchange. Considering these challenges and potential benefits, designing, and implementing blockchain solutions in the healthcare sector is essential.

### 5.6. Device-to-Device (D2D) Communications

D2D communication allows devices within range of each other to make direct contact. D2D communication can increase network spectrum efficiency, energy economy, transmission latency, traffic offloading, and core network congestion relief [99]. It can assist Healthcare 4.0 by enabling direct communication between nearby devices without the need for central network infrastructure.

### 5.7. Related Work

There are many proposals that consider developing robust Healthcare 4.0 systems. Such works are built based on previously introduced technologies. In this sub-section, we introduce the relevant works, and Table 12 summarizes the main features and the used key technologies for the recently developed Healthcare 4.0 and IoMT.

In [100], the authors developed a healthcare model based on ML for early and accurate prediction of different diseases. They used seven ML classification models, including support vector machine, neural network, decision tree, random forest (RF), naive algorithm, K-near neighbor, and adaptive reinforcement, to make predictions for nine deadly diseases, such as breast cancer, thyroid, and dermatology. The authors used sensitivity, the area under the curve, accuracy, and specificity as four measures of performance to evaluate the proposed model. The classifier has a maximum accuracy of 97.62 percent, a sensitivity of 99.67 percent, a specificity of 97.81 percent, and an area under the ROC curve of 99.32 percent. Doctors can detect the conditions early because of this new healthcare approach.

In [101], the authors identified the main parts of a dynamic cyber security architecture for protecting healthcare IoT infrastructure. They simulated the framework using evolutionary game theory and evaluated it. The simulation findings showed the defense’s optimal response to dynamic and adaptive foes. The authors suggested taking quantitative measurements for future work. Also, they suggested designing game simulations to fully understand and predict the evolutionary dynamics of adaptive assault and defense utilizing ML and evolutionary game theory.

In [102], the authors examined how medical big data and wearable IoT healthcare systems may be used to remotely monitor and care for COVID-19 patients who had been confirmed or suspected. They used information gathered from many sectors, including Accenture, Amwell, Deloitte, Ericsson ConsumerLab, Kyruus, Rockefeller Foundation, Syneos Health, and USAID, to conduct analyses and make estimates about the use of AI-driven biosensors in diagnosis, surveillance, and prevention during the COVID-19 pandemic. COVID-19 prevention, screening, and treatment have all benefitted from AI-powered diagnostic technologies.

In [103], the authors built an e-health model with multiple patient agent software instances with three layers: sensing, close processing, and remote processing. They described a possible 5G device configuration for the patient agent. The dedicated patient agent app could control the resources of individual 5G network slices. An investigation of the new e-health app’s performance indicated that it would use blockchain technology to handle health data in near-real time.

The research in [104] provided a road map for introducing IoT and AI into healthcare. The study investigates the future of healthcare and how AI would be combined with sensor-enabled IoT-based medical equipment. The groundwork, for future healthcare services, has been laid through a review of the literature on AI, ML, utilizing smart systems, and IoT in healthcare. The authors explained how a network of medical professionals might remotely monitor patients using wearables supported by the IoT and AI. The research stated that seamless integration between patients, doctors, and other healthcare professionals allows for timely, convenient healthcare delivery. The authors investigated how future smart healthcare technologies can facilitate cooperation amongst network partners in delivering continuous patient monitoring and care. A comprehensive framework was provided that aids medical professionals in delivering top-notch treatment to their patients. However, the study focused on the medical field.

In [105], the authors proposed a fog-computing architecture for processing sensor data that makes efficient use of shared resources. The model was built using publish/subscribe and distributed hash tables (DHT). The authors recommended architecture for efficient sharing and use of resources. The issues with processing data in the cloud have been fixed by introducing fog computing to Healthcare 4.0. Publish/subscribe and peer-to-peer (P2P) overlays are used in the proposed framework to satisfy the requirement for fast node discovery.

End-to-end security solutions are necessary to prevent and regulate health data breaches because of the sensitive nature of this data. Several authentication and authorization systems can protect private information gathered by IoT devices in Healthcare 4.0. The transport layer security (TLS) protocol aims to make information transmission more secure. Using this protocol, a user can avoid the issue of communications getting lost or switched around. 

A tricky flaw in the datagram transport layer security (DTLS) protocol is that an attacker might flood a server with ClientHello messages. With each new ClientHello message, the denial-of-service (DoS) attacker can gain more bandwidth and resources by establishing a new connection to the server. The authors in [106] developed an intelligent gateway-based authentication and authorization model to solve these issues. The proposed model keeps attackers and malicious users from accessing important physiological data. The authors used the Contiki Network Simulator to demonstrate the improved DTLS based on smart gateways. The packet loss ratio was used as a performance measure for the introduced work. The enhanced DTLS’s efficacy was evaluated by timing data transfers and handshakes.

The meaning of fog computing in the context of Healthcare 4.0 was examined in [107]. Fog computing has proven to be a successful answer to various healthcare concerns. The research used a framework that compared various healthcare technologies against present technology. Issues and challenges in healthcare have been examined, as well as health-related technologies. Fog computing has been regarded as a well-designed data processing solution that combines the benefits of the cloud. The work included case studies from rural locations, including ECG monitoring and diabetes management.

In [108], the authors introduced an AI-driven decentralized healthcare system that can access and verify IoT devices while guaranteeing PHR’s integrity and openness. The method was predicated on the idea of public blockchain networks and AI-enabled smart contracts. The framework also identified any malicious IoT nodes. An additional AI layer, based on rules, detected malicious nodes and made educated decisions by combining them with existing smart contracts. Request creation and registration times, energy usage per device, transaction throughput, and average latency in a real-world scenario were all measured to evaluate the effectiveness of the proposed framework. In order to increase the system’s dependability and create lighter algorithms to reduce energy and gas usage, further research on trustworthy AI utilizing the proposed framework could be carried out in the future.

In [109], the authors focused on the problems facing the healthcare system. They proposed a system architecture and an algorithm for access control policies for participants in the EHR system to ensure the confidentiality and security of patient information. A blockchain-based EHR sharing solution was also described. The suggested work eliminates the system’s single point of failure and central authority. Immutable ledger technology ensured system security by preventing users from altering the ledger. By adjusting parameters, including block size, block formation time, endorsement policy, and suggested optimization, for evaluating metrics, such as latency, throughput, and network security, the caliper was used to assess the effectiveness of the proposed system. The proposed system’s performance was increased by 1.75 times, and its latency was reduced by 1.5 times by optimizing the performance. This demonstrates the blockchain’s utility and importance in a variety of fields, demonstrating that it has the potential to be the next revolutionary technology to replace present healthcare systems.

In [110], the authors suggested a novel strategy that used Healthcare 4.0 to defend against central authority failure and implement a decentralized approach. The performance of the overall system and sub-systems were improved using a simulation-optimization technique. The suggested method was tested, confirmed, and validated using simulation and implementation. In order to evaluate the performance of the system and its dependent and independent variables, several parameters were used in simulation and implementation. The error in the implementation and simulation results varied from a minimum of 0.55 to a maximum of 4.24 due to variations in the environment and simulator limitations. This comparison analysis supported the validity of the proposed approach. The authors suggested expanding the work in the future to be implemented on various blockchain networks using various tools and techniques. In addition, there will be a comparison of current and future approaches.

## 6. Research Directions

The following research directions can assist the evolution of Healthcare 4.0 and IoMT. These research directions aim to address challenges, improve healthcare delivery, enhance patient outcomes, and maximize the benefits of IoMT and Healthcare 4.0.

(a) Emphasis on medical data analysis and management: There is great demand for developing robust data management strategies to handle the vast amount of data IoMT devices generate. This includes ensuring data security and privacy and using advanced analytics tools to derive valuable insights from the data. With the massive amount of data generated by IoMT devices, there is a need for advanced data analytics techniques and AI algorithms to extract meaningful insights. The research aims to develop predictive models, machine learning algorithms, and AI-driven decision support systems to improve patient outcomes, disease prevention, and healthcare operations.
(b) Security and privacy improvement: As healthcare systems become increasingly connected, ensuring the security and privacy of medical data is crucial. Research focuses on developing robust security measures, encryption techniques, and protocols to protect patient information and prevent unauthorized access. Data security is a primary concern for medical data due to the sensitivity of medical records. Encryption, secure networks, and access controls can help protect data from unauthorized access. Regular security audits and firmware updates are crucial in maintaining a secure environment. Researchers are interested in deploying decentralized schemes, e.g., blockchain, to assist medical data security. Also, lightweight security algorithms are a new direction to meet the energy requirements of medical sensors. With the vast amount of personal health data being collected, there is a risk of unauthorized sharing or use of this information. Implementing strong privacy policies, acquiring informed consent, and applying data anonymization techniques can help protect patient privacy. It is important to ensure compliance with relevant data protection regulations. Trust management techniques are a promising way introduced for achieving these requirements.
(c) Interoperability: IoMT devices and systems often come from different manufacturers and may use proprietary protocols, leading to interoperability challenges. Research focuses on developing standardized frameworks and protocols to ensure seamless communication, data exchange, and integration of IoMT devices and systems across healthcare settings.
(d) Edge computing solutions: IoMT generates a large volume of real-time data, which can be challenging to transmit and process in traditional cloud-based architectures. Researchers have been investigating the deployment of mobile edge computing and fog computing techniques to assist IoMT and Healthcare 4.0. This includes developing network structures based on edge units, proposing edge intelligence schemes to assist medical data, and developing offloading schemes that meet network requirements.
(e) Quality of experience improvement: Understanding the user experience, acceptance, and adoption of IoMT devices among healthcare professionals and patients is crucial. The research investigates usability, user interface design, and human factors considerations to ensure that IoMT technologies are user-friendly, efficient, and meet the needs of healthcare stakeholders.
(f) Ethical and legal considerations: There are ethical and legal implications for patient privacy, data ownership, consent, and accountability. Research explores the ethical and legal frameworks, guidelines, and policies necessary to govern the use of IoMT in healthcare settings, ensuring transparency, fairness, and adherence to ethical principles.

## 7. Ethical Implications of Healthcare 4.0

Healthcare 4.0 presents exciting opportunities for improving healthcare delivery, but it also raises ethical concerns related to discrimination, data privacy, and job displacement. It is crucial for stakeholders in the healthcare industry, including healthcare providers, policymakers, and technology developers, to collaborate and implement ethical frameworks that prioritize patient welfare, privacy, and fair access to healthcare services. By addressing these ethical implications proactively, the benefits of Healthcare 4.0 can be maximized while its potential drawbacks can be minimized.

**A.**  **Potential for discrimination against certain groups of people:**

Healthcare 4.0 relies heavily on digital technologies that raise concerns about the fair and equitable treatment of individuals. The following are some key concerns:

1. Data and algorithmic biasing: If the data used to train algorithms is biased or incomplete, the AI systems may make decisions that perpetuate existing healthcare disparities. For instance, if historical data disproportionately represents certain groups, the AI might provide less accurate or inadequate diagnoses and treatments for other underrepresented groups. Thus, if certain groups of people are underrepresented in the training data used to develop AI algorithms, the algorithms might not perform as accurately for those groups. This could lead to biased treatment recommendations or misdiagnoses [114]. In addition, if access to healthcare technologies or AI-based services is restricted based on socioeconomic status or geographic location, this could exacerbate existing healthcare disparities.
2. Privacy and consent: As digital technologies collect vast amounts of personal health data; individuals’ privacy becomes a major concern. Healthcare 4.0 should address many privacy and consent issues. Mitigating privacy risks and ensuring secure data management in Healthcare 4.0 systems can be achieved in several ways, as presented in Table 13. However, Table 14 presents the potential ways to ensure proper patient consent [115,116].
3. Health insurance discrimination: Health data could potentially be used by insurance companies to assess risk profiles and adjust premiums accordingly, disadvantaging individuals with specific health conditions or genetic predispositions.

**B.**  **Misuse of personal health data**

Healthcare 4.0 significantly increases the risk of personal health data being misused or mishandled. The full digitization of healthcare 4.0 systems increases the number of cyber criminals. Medical data will be targeted for malicious purposes. Moreover, the commodification of health data could lead to scenarios where private companies profit from selling or using patients’ health information for marketing or research purposes without adequate consent. To address these ethical implications, healthcare organizations and policymakers must prioritize cybersecurity measures to protect patient data from breaches and unauthorized access. There should be stringent regulations in place governing the use of personal health data, ensuring that data is only used for legitimate purposes and with explicit patient consent [117].

**C.**  **Job loss of healthcare workers**

The integration of digital technologies in Healthcare 4.0 will lead to the automation of many tasks, which raises concerns about potential job displacement for healthcare workers. Healthcare institutions should focus on workforce planning and invest in upskilling and reskilling programs for employees to mitigate this impact. Moreover, policymakers can implement regulations that balance automation with maintaining high-quality patient care, ensuring that technology complements human capabilities rather than replacing them entirely [118].

## 8. Conclusions

IoMT and Healthcare 4.0 have the potential to revolutionize the healthcare sector. This work discussed the main features of the Healthcare 4.0 paradigm and how it will shape the future of medical services from the hardware and communication technology point of view. The current challenges of Healthcare 4.0 systems and the main key enabling technologies that can solve these challenges were presented. The recent advances and future directions of Healthcare 4.0 and IoMT are presented, providing a way for researchers to go through. By leveraging technology, healthcare systems can become more proactive, personalized, and efficient. However, data security, privacy, and implementation challenges should also be addressed to maximize the benefits of these advancements.

## Figures and Tables

**Table 1:** Main characteristics of WBAN.

Feature	WBAN
Reliability	It refers to the ability of WBAN to consistently and accurately transmit and deliver data from medical sensors to the intended destinations without loss, or corruption. The reliability of WBAN should be higher than that of regular sensor networks due to the importance of medical data.
Energy	Deployed devices in WBAN are battery-operated, which puts many constraints on the network design. Batteries may be easily swapped out in the not implanted sensor; however, it is complicated for sensors implanted surgically in the patient’s body.
Human-centric	WBANs are attached directly to body parts in order to capture data as a collection of sensed information. The human body is both exceedingly sensitive and receptive to these sensors. Therefore, these sensors must be safe and simple for the human body to accept.
Availability	WBANs should support high availability since medical data are critical. Links between devices and the network sink should always be available.
Mobility	WBAN should support moderate-to-high mobility since devices are attached to humans. Based on daily life activities, the mobility of sensor nodes changes, and thus the network should provide stable performance for different mobilities.
Scalability	Unlike wireless sensor networks, WBANs are small-scale networks dedicated to specific medical applications. Depending on their size, up to fifteen sensors are affixed in, on, or around the user’s body.
Deployment	Numerous sensors are connected directly to exit sites and gateways in these networks, localizing sensors to form a dense network. Unlike WSNs, WBANs have no constraints in terms of dense deployment scenarios. However, WBANs suffer from interferences between nearby WBANs, which are limited in large-scale WSNs.
Topology	WBAN does not have constraints on network topology, and thus there is no fixed topology associated with it. Dynamic, efficient topologies, including stars, meshes, hybrids, and clusters, are all useful in WBANs.
Bandwidth	The bandwidth of WBANs varies depending on the specific technology and protocol used. Short-range communication technologies, including Bluetooth, Zigbee, and Wi-Fi, are commonly used for WBANs; however, battery usage is challenging with such interfaces. Therefore, the bandwidth of WBANs can range from hundreds of kbps up to a few Mbps, depending on the deployed technology and protocol.

**Table 2:** Common market available wearable healthcare watches.

Commercial Name	Structure	Technical Specification	Biological Measurement
Apple Watch [34]	Optical heart rate sensor Electric heart rate sensor Blood oxygen sensors Altimeter GPS Compass Microphone Tactile digital crown	(a) Connectivity	Records sleep hours. Measures blood oxygen levels. Monitors PA and HR
		Wi-Fi (IEEE 802.11b/g/n) 2.4 GHz and 5 GHz LTE (Licensed spectrum) Bluetooth 5.3	
		(b) Display	
		Retina LTPO OLED display Brightness up to 2,000 nits	
		(c) Power	
		USB-C magnetic fast charging Built-in lithium-ion rechargeable battery	
		(d) Processor (S7 Sip Dual Core)	
Samsung Galaxy Watch-5 [35]	Bioactive sensor Temperature sensor Geomagnetic sensor Electrical heart sensor Barometer sensor Optical heart rate sensor Light sensor Bioelectrical impedance Microphone GPS	(a) Connectivity	Body composition analysis Monitoring blood oxygen levels Sleep tracking Monitoring heart rate Guided workouts Irregular heartbeat detection Snore detection
		LTE Bluetooth 5.2 Wi-Fi (IEEE 802.11a/b/g/n) 2.4 + 5 GHz NFC	
		(b) Display	
		Sapphire crystal 1.4” (34.6 mm) 450 × 450 Super AMOLED Full color	
		(c) Power	
		Fast charging (WPC-based wireless charging) Battery-590 mAh	
		(d) Processor (Exynos W920 Dual-Core 1.18 GHz)	
Huawei Watch GT, Huawei Band [36]	Optical heart rate sensor Accelerometer sensor Capacitive sensor Ambient light sensor Gyroscope sensor Air pressure sensor Geomagnetic sensor Microphone GPS	(a) Connectivity	Tracks sleep Menstrual cycle and stress Monitors HR 24/7 Monitoring SpO2
		Bluetooth 4.2 LTE GLONASS, and Galileo positioning Wi-Fi (IEEE 802.11a/b/g/n) 2.4 + 5 GHz NFC	
		(b) Display	
		Type: OLED Size: 1.39 inches Resolution: 454 × 454 pixels (~326 ppi density)	
		(c) Power	
		Li-Ion 420 mAh	
		(d) Processor ARM Cortex-M4	
Fitbit Versa [37]	Optical heart rate sensor Skin temperature sensor Three-axis accelerometer Infrared and red-light sensors Microphone GPS Vibration motor Altimeter sensor	(a) Connectivity	Examines breathing rate. Skin temperature changes Measures SpO2. Sleep phases. Sleep quality. Tracks body activities. Tracks calories burned. Measures health stress, emotions, and breathing exercises. Heart rate, resting heart rate, and cardiovascular fitness.
		Wi-Fi (IEEE 802.11a/b/g/n) 2.4 + 5 GHz Bluetooth NFC LTE GLONASS	
		(b) Display	
		Size: 1.58 inches Resolution: 336 × 336 pixels	
		(c) Power	
		Li-Ion 420 mAh	
		(d) Processor ARM Cortex M4F	
Withings [38]	Accelerometer Altimeter ECG with 3 electrodes Microphone GPS	(a) Connectivity	Records ECG readings
		Bluetooth LTE	
		(b) Display	
		13.8 mm PMOLED	
		(c) Power	
		Li-Ion 420 mAh	
Xiaomi Mi Smart Band 5 [39]	Accelerometer Digital MEMS microphone Gyroscope SpO2 sensor GPS Heart rate sensor Barometer Proximity sensor	(a) Connectivity	Monitors the health of women. Monitors sleep (deep sleep, light sleep, REM sleep, naps). Monitors heart rate. Tracks stress.
		Bluetooth 5.0 NFC	
		(b) Display	
		AMOLED color touchscreen Size: 1.1 inches Resolution: 126 × 294 (450 nits) Glass with 2.5D reinforcement and AF coating	
		(c) Power	
		125 mAh 14-day battery life	
		(d) Processor (Dialog DA14697)	

**Table 3:** The main features for selecting a pulse rate sensor.

Feature	Selection Criteria
Accuracy and reliability	The sensor should provide accurate and reliable pulse rate readings, as any inaccuracies can lead to incorrect diagnosis or treatment.
Signal quality	The sensor should produce a clear, high-quality signal that medical professionals or automated systems can easily interpret.
Comfort and usability	Consider the comfort of the sensor for the patient, as they may need to wear it for an extended period. Look for sensors that are non-intrusive, lightweight, and easy to use.
Sensing technology	Different sensing technologies are available, such as photoplethysmography (PPG), electrocardiography (ECG), or ballistocardiograph (BCG). Based on the specific needs of the application, suitable technology is chosen.
Compatibility	Ensure the sensor is compatible with the intended medical device or system. This includes interface compatibility, data communication protocols, and power requirements.
Environmental considerations	Evaluate the intended use environment, such as temperature, moisture, or electromagnetic interference, to ensure the sensor can withstand and perform well in such conditions.
Cost	Consider the cost implications, as medical sensors can vary in price. Balance the required features and performance with the available budget.
Regulatory compliance	Ensure that the selected sensor meets the necessary medical device regulations and certifications, such as FDA approval or CE marking, depending on the use region.

**Table 4:** Common market available IoT medical sensors.

Category	Available Market	Features and Technical Specification
Blood oxygen sensors	MAX30100 Heart Rate Oxygen Pulse Sensor [62] 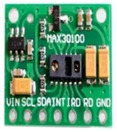	Operational voltage spans the range of 1.8 V and 3.3 V. The temperature fluctuates from −40 °C to +85 °C. This module may be utilized with interrupts, enabling a variety of sources such as SpO2data ready, power ready, temperature ready, and heart rate data ready. Temperature precision is 1 °C. The peak wavelength for infrared LEDs spans from 870 to 900 nm. Ambient light cancellation is integrated. It uses an ADC of a 14-bit resolution. It deploys a red LED with peak wavelengths between 650 and 670 nm. Its data output speed is rapid. 20 mA is the input current. Sample rate and LED current may be modified to save power. It deploys power-saving features such as programmable sample rate and LED current. It has an extremely low shutdown current (typically 0.7 μA).
	MAX30102 Heart Rate Oxygen Pulse Sensor [63] 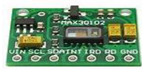	Operating temperature range: −40 °C to +85 °C. It deploys an LED reflective solution. It deploys power-saving features such as programmable sample rate and LED current. It achieves a high signal-to-noise ratio (SNR). It uses a low-power monitor (less than 1 mW). Strong resistance to motion artifacts. It has an extremely low shutdown current (typically 0.7 μA). It deploys a tiny 14-pin optical module.
Blood pressure sensors	Blood pressure sensor [64] 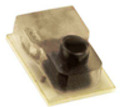	The temperature fluctuates from 5 to 40 °C. Able to work with automated assembly. It deploys a Gel liquid isolation. It has a 5 V/V/mmHg sensitivity. It has a compact size and achieves dependable results. It has a 1% replacement accuracy. It uses a barrier of dielectric Gel.
	Pressure Sensor MPS20N0040D-S [65] 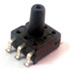	Range of pressure: 0–580 psi. Power source: 5 V DC or 1 mA of continuous current. Temperature range: −40 to 85 °C (−40 to +185 °F). It uses a 4 to 6 KΩ bridge resistance. It uses a 4–6 KΩ input impedance. It deploys a 5 V DC power supply. It has a 4 to 6 K output impedance.
Heart rate sensors	Pulse Sensor [66] 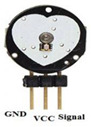	It operates at a voltage of +5 V/+3.3 V. Light source: super red LEDs at 660 nm. This sensor measures the heart rate and biometric pulse rate. It deploys a noise cancellation circuit. It deploys a plug-and-play model. It uses integral amplifiers. 100 mA is the maximum current. VCC is +5 v DC. 4 mA is the current consumption.
	AD8232 ECG Sensor [67] 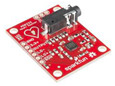	It uses a three-pole low pass filter (LPF) with adjustable gain. The operating range of the supply is 2 to 3.5 V. Right leg drive (RLD) amplifier included. It enables high signal gain by utilizing DC blocking. It uses a two-pole versatility high pass filter (HPF). Only lead ECG is incorporated into the front end as a whole. There are 2 or 3 electrode combinations.
	Heart rate sensor IR [68] 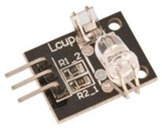	The pulse monitor operates as follows: the phototransistor is on the opposite side of the finger from the LED, which is turned on one side of the finger. It deploys a Ky-039 heartbeat sensing module. This heartbeat detection module detects the pulse of the finger using a strong infrared (IR) lead and a phototransistor, a red LED flashes with each pulse. To detect pulsation in fingers, it uses an optical transistor and an IR LED. When blood flows through the finger, the phototransistor’s resistance will change somewhat, allowing for the collection of the flux emitted.
Blood glucose sensors	Wireless glucose meter [69] 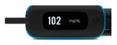	It measures blood glucose in mg/dL. It has an integrated LED display. It deploys a Bluetooth interface. It achieves an accuracy of 99%. It uses a 3 V battery.
ECG	ECG/EKG Monitoring [70] 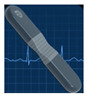	It enables real-time tracking. It deploys AI to assist signal analysis. Detect many heart abnormalities, including atrial flutter and fibrillation. It is a lightweight rechargeable device, which facilitates individual use. It supports various operating systems. Power source: 3.7 V DC. It achieves a maximum error of ±10%.

**Table 5:** Energy challenges with Healthcare 4.0.

Challenge	Issues with Healthcare 4.0
High energy consumption technologies	Healthcare 4.0 systems incorporate a wide range of energy-intensive technologies, such as medical imaging equipment, laboratory instruments, and data centers. The cumulative energy demand can be significant, leading to increased operational costs and environmental impact.
Battery life of medical devices	Healthcare 4.0 relies on battery-operated medical sensors and wearable devices for real-time communication and access to patient information. Limited battery life can be a concern during extended shifts or emergencies, affecting the continuity of care. Modern communication networks, e.g., IoMT, demands ten years of battery life.
Energy efficiency of medical devices	Medical devices may lack energy-efficient designs. As these devices remain in use for extended periods, their higher energy consumption contributes to overall energy challenges in healthcare settings.
Power management	The proliferation of IoMT devices in Healthcare 4.0 requires careful power management. Frequent battery replacements or recharging protocols can be burdensome and impact device utilization.
Renewable energy integration	Incorporating renewable energy sources, e.g., solar, into Healthcare 4.0 facilities can help reduce reliance on traditional energy sources. However, implementation complexities, including wireless power transfer challenges, present challenges.

**Table 6:** Massive data challenges with Healthcare 4.0.

Challenge	Issues with Healthcare 4.0
Data volume	Healthcare 4.0 generates vast volumes of data from massive deployed medical devices. Managing and processing such large datasets can strain existing storage and computing resources.
Data diversity	Healthcare data comes in various formats, including structured data (e.g., EHRs), unstructured data (e.g., medical imaging), and streaming data from IoMT devices. Integrating and analyzing this diverse data is complex and requires advanced data processing techniques.
Data generation rate	The speed at which data is generated and needs to be processed in real-time can pose traditional data processing systems. Real-time analysis is critical for immediate clinical decisions and timely interventions.
Data analytics and insights	Extracting meaningful insights from large and complex healthcare datasets requires sophisticated data analytics tools and expertise. Analyzing data effectively can be resource-intensive and time-consuming.
Data storing and archiving	Storing and managing historical healthcare data can be challenging due to its volume and the need for long-term retention for research, legal, and compliance purposes.

**Table 7:** Summary of Healthcare 4.0 challenges.

Challenges	Issues with Healthcare 4.0	Key Solutions
Energy	Healthcare 4.0 systems should support a ten-year battery life of medical sensors. Thus, communication networks and protocols used for such systems should meet this demand.	Energy-efficient network algorithms Edge computing Quantum computing
Bandwidth	With the growing use of telemedicine and other remote healthcare services, communication networks must be able to facilitate high-quality video and audio streaming with sufficient bandwidth. Healthcare 4.0 faces significant difficulty in allocating resources optimally for vital medical traffic.	Edge computing 5G communications D2D
Network availability and reliability	Achieving ultra-high availability and reliability is a challenge that needs to be resolved by introducing novel technologies.	5G communications D2D Edge computing Edge intelligence
End-to-end latency	A part of the Healthcare 4.0 services are ultra-low latency services that require an end-to-end latency of 5-1 msec for the communicated data. This puts many constraints on the development of such networks.	Edge computing Edge intelligence Software-defined networking
Security	The required security level of Healthcare 4.0 applications cannot be achieved by the existing traditional schemes. Also, traditional security protocols cannot meet the energy demands of Healthcare 4.0.	Trust management Blockchain Quantum encryption
Scalability	The designed networks and protocols for Healthcare 4.0 applications should support high scalability since medical devices are growing rapidly.	5G communications D2D Edge computing
Massive data	The massive amount of network traffic due to numerous devices deployed in the network should be managed using robust methods to avoid network failure.	Big data D2D Edge computing Blockchain

**Table 8:** Benefits of 5G communications in Healthcare 4.0.

Benefit	Role in Healthcare 4.0
Faster (ultra-low latency) communications	With its high data transfer speeds and low latency, 5G can enhance real-time communications between healthcare professionals, allowing for faster transmission of critical patient data, remote consultations, and telemedicine services.
Ultra-high reliability	Ultra-high reliability is one of the key characteristics of 5G communications. Relying on 5G’s ultra-reliable connections ensures seamless communication between healthcare professionals and support staff. This provides a robust foundation for Healthcare 4.0, enabling transformative advancements in remote care, real-time monitoring, telemedicine, and precision medicine.
Ultra-high availability	The ultra-high availability of 5G communications allows Healthcare 4.0 systems to consistently deliver services with very minimal downtime or disruptions. It emphasizes the network’s ability to remain operational and accessible to users and applications for an extended period. This is an important issue with medical services due to the emergency of most medical services.
High scalability	Scalability is a critical issue with Healthcare 4.0 due to the evolution and growth of medical devices and technologies. 5G communications can significantly increase the scalability of Healthcare 4.0 by enabling seamless integration and expansion of advanced technologies and healthcare services.
Integration with the Internet infrastructure	5G communications can assist in integrating medical sensors into the Internet and supporting remote services via a 5G cellular interface. Many remote Healthcare 4.0 applications have problems with the appropriate communication interface between devices and the Internet. These problems can be solved using 5G communications.
Extended coverage	By utilizing 5G technology, healthcare providers can extend their reach to remote and underserved areas.
Data security and privacy	The implementation of 5G prioritizes robust security measures to protect patient privacy and prevent data breaches.

**Table 9:** Limitations of 5G communications in Healthcare 4.0.

Implication	Issue with Healthcare 4.0
Interference	The high-frequency spectrum used by 5G can be susceptible to interference from physical objects, such as walls or medical equipment. Ensuring reliable coverage and minimizing potential signal disruptions is crucial.
Cost	The deployment of 5G networks and associated hardware can be expensive. Healthcare organizations need to assess the cost implications and potential return on investment when considering 5G implementation.
Infrastructure	5G networks rely on a dense infrastructure of small cells and base stations. Implementing this infrastructure within healthcare facilities may require significant infrastructure upgrades.
Regulatory	The implementation of 5G in healthcare may involve compliance with specific regulations governing data privacy, patient consent, and network safety. Adhering to these regulations is essential to avoid legal and ethical issues.
Public fears and beliefs	While 5G technology offers numerous benefits, some people have expressed concerns and fears about its deployment, especially in medical services. One of the primary fears revolves around potential health risks associated with increased exposure to electromagnetic radiation. Some individuals worry that the higher frequency and intensity of 5G signals might have adverse effects on human health. It is essential to address these fears through rigorous scientific research, transparent communication, and robust regulatory measures.

**Table 10:** Benefits of cloud computing to Healthcare 4.0 and IoMT.

Benefit	How It Assists Healthcare 4.0 and IoMT
Storage and accessibility	Cloud computing allows medical professionals to securely store and access patient data from anywhere, anytime, using any internet-connected device. This streamlines data management eliminates physical storage constraints and facilitates efficient collaboration among healthcare providers.
Scalability	Cloud platforms can scale computing resources based on demand. This is particularly beneficial for medical professionals dealing with data-intensive processes or situations requiring rapid scaling, including during emergencies.
Data backup	Cloud services typically offer robust backup and recovery capabilities, ensuring data resilience and minimizing the risk of data loss in case of local system failures or natural disasters. This feature is vital to save patient information and maintain continuity of care.
Enhanced collaboration	Cloud computing enables real-time collaboration between medical professionals, allowing them to securely share patient records, test results, and treatment plans. It also supports telemedicine by providing a platform for remote consultations, remote monitoring, and virtual patient care.
Cost-effectiveness	Cloud computing eliminates the need for extensive on-premises infrastructure, IT hardware, and maintenance costs. It offers a cost-efficient business model, allowing medical professionals to scale resources as needed, optimizing cost-efficiency.
Security and compliance	Cloud providers invest heavily in advanced security measures, encryption techniques, and regulatory compliance standards. By leveraging cloud services, medical professionals can benefit from robust security protocols and ensure adherence to healthcare privacy regulations.

**Table 11:** Benefits of distributed edge computing to Healthcare 4.0 and IoMT.

Benefit	How It Assists Healthcare 4.0 and IoMT
Reducing latency	Processing data at the edge, near the patient or device, minimizes the time it takes for data to travel to a central location. This reduced latency is critical for real-time health monitoring, emergency responses, and critical care scenarios.
Enhanced privacy and security	With distributed edge computing, sensitive healthcare data can be processed locally, reducing the need for transmitting it over the Internet or to centralized servers. This approach improves data privacy and security, minimizing the chances of unauthorized access or data breaches.
Bandwidth optimization	Healthcare 4.0 and IoMT generate massive amounts of data, which can strain network bandwidth if transmitted to the cloud for processing. By leveraging distributed edge computing, data can be processed, filtered, and aggregated at the edge, minimizing the amount of data that needs to be transmitted over the network. This optimization improves overall network efficiency.
Reliable connectivity	In healthcare, consistent data connectivity is vital. The ultimate connectivity requirements of Healthcare 4.0 and IoMT can be assisted by distributed computing. Edge computing allows IoMT devices to continue functioning even during intermittent or unstable network connections. By storing critical data locally, healthcare systems can continue to operate even if the network goes down temporarily.
Real-time analytics and decision-making	Time-sensitive healthcare applications depend on real-time data analysis and prompt decision-making. Distributed edge computing enables data processing locally, facilitating instant insights and reducing response times. This is essential for monitoring patient vitals, detecting anomalies, and triggering timely interventions.
Cost efficiency	Offloading computation and storage to local edge servers can reduce the need for expensive cloud infrastructure and bandwidth. Also, it simplifies the requirements of the core network. This can lower operational costs associated with data transmission and storage, making healthcare services more affordable.
Offline assistance	In some healthcare scenarios, network connectivity may not always be reliable or available. Distributed edge computing allows health systems to function offline or with intermittent connectivity by processing data locally. This ensures uninterrupted patient care and data collection even in challenging network conditions.

**Table 12:** Summary of the main features and the used key technologies of the considered related works.

Ref.	Key Enabling Technology							KPI	Medical Application
	Cloud Com.	Block.	AI	Big Data	5G	Fog	MEC		
[100]	×	×	√	×	×	×	×	Accuracy Sensitivity Specificity Area under the curve	Diseases prediction
[101]	×	×	×	×	×	×	×	Average utility against common attacks Defense rate	Security of medical data
[102]	√	×	√	√	×	×	×	-	Remote monitoring and caring
[103]	×	×	√	√	√	√	×	Energy Throughput Reliability Traffic overhead	Healthcare
[104]	×	×	√	×	×	×	×	-	Monitoring routine activities
[105]	√	×	×	√	×	√	×	-	Healthcare monitoring
[106]	×	×	×	×	×	×	×	Packet loss Handshake time	Security of medical data
[107]	√	×	×	×	×	√	×	-	General
[108]	×	×	√	×	×	×	×	Latency Energy Throughput	Remote monitoring
[109]	×	√	×	×	×	×	×	Latency Security Throughput Resources utilization	Security of medical data
[110]	√	√	×	×	×	×	×	Latency Security	Security of medical data
[111]	√	×	×	×	×	√	×	-	General
[112]	×	×	√	√	×	√	×	-	General
[113]	×	√	×	×	×	×	×	Encryption time Decryption time Execution time	Security of medical data

**Table 13:** Potential solutions to achieve privacy issues in Healthcare 4.0.

Issue	Potential Solution
Implement strong access controls	Strict user authentication mechanisms, e.g., multi-factor authentication, should be enforced to prevent unauthorized access to sensitive healthcare data.
Encrypt sensitive data	Robust encryption algorithms to encrypt data and save patient information from being accessed or manipulated by unauthorized individuals.
Regularly update and patch systems	Keeping software, operating systems, and network infrastructure up to date with the latest security patches.
Conduct thorough risk assessments	Regularly assess the potential risks and vulnerabilities to the data management systems. This includes identifying potential threats, analyzing their impact, and addressing any identified risks through appropriate mitigation measures.
Follow regulatory frameworks and compliance standards	Following regulations, e.g., HIPAA and GDPR, ensure data handling in a legally compliant and secure manner.
Implement data anonymization techniques	Health data can be de-identified or anonymized to minimize the risk of re-identifying individuals.
Monitor and detect security incidents	Implement robust intrusion detection systems and security monitoring tools to detect and respond to any unauthorized access or suspicious activities.
Establish data breach response plans	Preparing and testing an incident response plan can minimize the impact of a data breach or security incident. This plan should outline steps to be taken, communication protocols, and strategies for containment and recovery.

**Table 14:** Potential solutions to ensure data consent in Healthcare 4.0.

Issue	Potential Solution
Digital consent forms	Implementing digital consent forms that patients can read and sign electronically is a vital solution. These forms should clearly explain the purpose, risks, and benefits of the proposed treatment or procedure, allowing patients to make informed decisions. The system can then store and record these consents securely.
Two-factor authentication	Using two-factor authentication methods, such as biometrics or unique codes sent to the patient’s mobile device, to verify the identity of the patient before consenting to any procedure ensures that only authorized individuals can provide consent.
Consent management systems (CMS)	Implementing CMS allows healthcare providers to track and manage patient consent throughout the care continuum. CMS automate consent processes, track consent status, and ensure compliance with legal and regulatory requirements.
Audit trails	Maintaining robust audit trails that record all consent-related activities, including when consent was obtained, who obtained it, and any subsequent updates or withdrawals of consent, helps establish a transparent record of patient consent.
Education and communication	Ensuring patients are educated about their rights and the importance of informed consent is critical. Healthcare 4.0 systems can incorporate patient portals, interactive interfaces, and educational materials to facilitate patient understanding and engagement.

## Data Availability

Not applicable.
